# Self-reported frequency and impact of hypoglycaemic events in insulin-treated diabetic patients in Austria

**DOI:** 10.1007/s00508-014-0626-1

**Published:** 2014-11-25

**Authors:** Raimund Weitgasser, Sandra Lopes

**Affiliations:** 1 Department of Internal Medicine, Diakonissen Hospital Salzburg, Salzburg, Austria; 2 Paracelsus Medical University Salzburg, Salzburg, Austria; 3 Novo Nordisk Scandinavia AB, Region Danmark, Arne Jacobsens Allé 17, 9. sal, 2300 Copenhagen, Denmark

**Keywords:** Diabetes mellitus, Hypoglycaemia, Insulin, Quality of life, Diabetes mellitus, Hypoglykämie, Insulin, Lebensqualität

## Abstract

**Background:**

Hypoglycaemia is a common side effect of insulin therapy and presents a barrier to diabetes management, however, limited data exist on the real-world frequency of events. We investigated the self-reported rates of non-severe and severe hypoglycaemic events in Austria. We also explored hypoglycaemia awareness, patient–physician communication and the health-related and economic impact of events.

**Methods:**

People with Type-1 or insulin-treated Type-2 diabetes > 15 years of age completed up to 4 questionnaires (weekly intervals). Non-severe hypoglycaemic events were defined by requiring no assistance while severe hypoglycaemic events need help from a third party.

**Results:**

Overall, 553 respondents (40 % Type-1, 60 % Type-2) enrolled, providing a total of 1,773 patient-weeks. The mean annual non-severe event frequencies were 85 for Type-1 and 15–28 for Type-2 (depending on insulin regimen). In respondents who experienced ≥ 1 non-severe event in the study period, annual rates were 18 % higher in Type-1 and 77 % higher in Type-2. The proportion of respondents reporting ‘awareness’ of hypoglycaemic symptoms was 48 % for Type-1 and 43–61 % for Type-2 respondents. The proportion of respondents who rarely/never inform their physician of hypoglycaemic events was 67 % (Type-1) and 43–53 % (Type-2). The most commonly reported health-related impacts were tiredness/fatigue (58 % of events) and reduced alertness (41 % of events).

**Conclusion:**

Non-severe hypoglycaemic events are common in Type-1 and insulin-treated Type-2 diabetes patients in Austria. There may be subgroups of patients who are predisposed to higher rates of non-severe events. Even non-severe events have a negative impact on physical and emotional well-being.

## Abstract (de)

**Grundlagen:**

Hypoglykämien sind ein häufiger Nebeneffekt der Insulintherapie und stellen eine Limitation im Diabetesmanagement dar, dennoch gibt es nur wenig Daten zu solchen Ereignissen im Alltag. Wir haben deshalb die Häufigkeit von selbst beobachteten nicht-schweren und schweren Hypoglykämien in Österreich untersucht. Ebenfalls untersucht wurden Hyoglykämiewahrnehmung, Patient-Arzt Kommunikation und gesundheitliche sowie ökonomische Auswirkungen von Hypoglykämien.

**Methodik:**

Personen mit Typ 1 oder insulin-behandeltem Typ 2 Diabetes ( > 15 Lj.) füllten in wöchentlichen Abständen bis zu 4 Fragebögen aus. Nicht-schwere Hypoglykämien (NSHE) wurden als solche ohne, schwere als solche mit von außen benötigter Hilfe definiert.

**Ergebnisse:**

Der Fragebogenrücklauf belief sich auf 533 Personen (40 % Typ 1, 60 % Typ 2), insgesamt 1.773 Patientenwochen entsprechend. Die durchschnittliche jährliche NSHE-Häufigkeit betrug 85 bei Typ 1 und 15–28 (abhängig von der Art des Insulinregimes) bei Typ 2. Bei Patienten, die ≥ 1 NSHE während der Studiendauer hatten, war die jährliche Hypoglykämierate bei Typ 1 um 18 %, bei Typ 2 um 77 % erhöht. Der Anteil der Patienten mit „Wahrnehmung“ hypoglykämischer Symptome lag bei 48 % für Typ 1 und 43–61 % für Typ 2. Der Anteil der Patienten, die selten/nie ihren Arzt über Hypoglykämien informieren, war 67 % bei Typ 1 und 43–53 % bei Typ 2.

Die am häufigsten berichteten gesundheitsbezogenen Auswirkungen waren Müdigkeit/Erschöpfung (58 % der NSHE) und eingeschränkte Aufmerksamkeit (41 % der NSHE).

**Schlussfolgerung:**

NSHE sind bei Typ 1 und insulin-behandelten Typ 2 Diabetespatienten in Österreich häufig. Subgruppen von Patienten mit einer Prädisposition für höhere Raten von NSHE könnten definiert werden. Selbst NSHE haben einen negativen Effekt auf das körperliche und emotionale Wohlbefinden.

## Introduction

According to the International Diabetes Federation (IDF) more than 56 million adults in Europe have been diagnosed with diabetes mellitus (2013 estimate), corresponding to a prevalence of 6.8 % [1]. The prevalence in Austria is slightly higher, at 8–9 % [2].

A fundamental goal in the management of patients with diabetes is the maintenance of normoglycaemia, often through the use of insulin [3]. However, intensification of insulin therapy can increase the incidence of hypoglycaemia; the most common and unpredictable side effect of insulin treatment [4]. Hypoglycaemia can be defined as either non-severe or severe according to whether a patient can manage the event alone or requires third party assistance, respectively [5, 6]. Non-severe hypoglycaemic events (NSHEs), which account for 88–98 % of all events [7, 8], are associated with a negative impact on health-related quality of life, healthcare resource use and work productivity [7, 9, 10].

Hypoglycaemia presents a significant barrier to optimal diabetes management as fear of hypoglycaemic events may cause exaggerated avoidance behaviour and consequently sub-optimal insulin therapy and glycaemic control [11, 12]. Therefore, diabetes education has a critical role in diabetes management, and all diabetes patients in Austria are offered structured diabetes education, which aims to minimise the risk of diabetes-related complications and premature mortality. For Type 2 patients, a Disease Management Programme (DMP) for regular diabetes care by GPs was introduced in 2007 [13]. However, only 15–20 % of Type 2 patients have enrolled in the programme, demonstrating an ongoing need for improved patient engagement in the management of their diabetes. Alongside patients, all physicians who care for people with diabetes are expected to attend the programme [13].

In Europe, data on the frequency of hypoglycaemia outside of clinical trial settings are limited and varied. The majority of literature focuses on Type 1 diabetes and the frequency of severe hypoglycaemic events (SHE). Four European studies have reported real-world estimates of NSHE rates [4, 8, 10, 14], however there are no data specific to the Austrian setting. The results of previous studies vary according to their definition of hypoglycaemic events, methods of data collection, and country coverage. Therefore there is a clear need to gain a better understanding of the patient perspective on the burden of hypoglycaemia, and their communication with healthcare professionals (HCPs) on the subject.

This paper reports the frequency of self-reported NSHEs and SHEs in people with Type 1 and insulin-treated Type 2 diabetes mellitus (T1DM and T2DM) in Austria. Additionally, levels of impaired hypoglycaemia awareness, patient–physician communication of hypoglycaemic events and the health-related effects and economic impact of NSHEs are reported.

## Patients, materials and methods

A full description of the methodology for this study has been previously described by Östenson et al., who investigated self-reported NSHE rates across seven Northern and Central European countries (Austria, Denmark, Finland, Norway, Sweden, Switzerland and the Netherlands) [15, 16].

The questionnaire-based survey was conducted in Austria between February and May 2012 People over the age of 15 with a T1DM or T2DM diagnosis receiving insulin were recruited using existing large online panels that provided a representative sample of the general diabetes population based on age, gender and other demographic characteristics. Respondents with T2DM were divided into three subgroups based on their insulin regimen: long acting-insulin only (basal only therapy; T2BOT), short and long acting insulin (basal-bolus; T2BB) or other insulin regimens (e.g. premix; T2O).

A small incentive was offered for completion of the questionnaire (approximately € 5–10 in total), in line with current market research guidelines and to ensure there was no undue incentive to participate. Questionnaires were completed anonymously in accordance to the regulations and practice of market research governing bodies European Society for Opinion and Marketing Research (ESOMAR) [17] and European Pharmaceutical Market Research Association (EphMRA) [18].

Participants were invited to complete four questionnaires over four consecutive weeks. The first questionnaire collected information on respondent demographics, awareness of hypoglycaemic symptoms, communication of hypoglycaemic events with HCPs, frequency of NSHEs in the previous seven days, and the number of SHEs in the past year. The subsequent three questionnaires recorded only the frequency of NSHEs in the preceding seven days, and the impact of the most recent event. Data collected on the impact of hypoglycaemia included changes in respondent well-being, work productivity and healthcare resource use. Weekly NSHE frequencies were calculated using data from all participants completing at least one questionnaire (wave), with annual frequency calculated using the mean weekly event frequency multiplied by 52. NSHE frequencies are also presented for only those respondents who experienced a NSHE during the recall report.

The classification system for awareness of hypoglycaemia was based on a prospectively validated study by Pedersen-Bjergaard et al. [19]. Any respondent who answered ‘sometimes’ or ‘never’ to the question ‘can you feel when your blood sugar is low?’ was assigned as being unaware of hypoglycaemia, those who answered ‘usually’ as having impaired awareness and those who answered ‘always’ deemed to be aware.

Standard descriptive methods (means/percentage and standard deviations) were used to report results. Comparisons of NSHE frequencies according to respondent awareness and patient–physician communication were performed using t-tests with an employed significance level of *p* < 0.05.

## Results

Overall 553 respondents completed the first wave, with 82 %, 72 % and 67 % completing waves 2, 3 and 4, respectively. This gives a total of 1,773 patient-week records in Austria. Patient demographics are shown in Table 1. In total, 40 % of respondents had T1DM and 60 % of respondents had T2DM.

**Table 1:** Respondent demographics

	Type 1	Type 2
Number of respondents, N (%)	222 (40 %)	331 (60 %)
Age, mean (SD)	44.5 (14.6)	62.8 (11.9)
Gender, female, N (%)	113 (51 %)	137 (41 %)
*Marital status, N (%)*		
Single	61 (27 %)	78 (24 %)
Married	109 (49 %)	211 (64 %)
Partner	52 (23 %)	42 (13 %)
*Living arrangements, N (%)*		
Alone	34 (15 %)	74 (22 %)
With others	188 (85 %)	257 (78 %)
Active employment, N (%)	143 (64 %)	74 (22 %)
*Education, N (%)*		
Primary school	67 (30 %)	159 (48 %)
High school	101 (46 %)	137 (41 %)
University	48 (22 %)	28 (8 %)
Other	6 (3 %)	7 (2 %)
BMI, mean (SD)	25.5 (4.8)	29.9 (5.7)
*Diabetes duration, N (%)*		
Mean years (SD)	20.2 (13.5)	16.4 (10.3)
< 2 years	7 (3 %)	3 (1 %)
2–5 years	21 (10 %)	37 (12 %)
5–9 years	30 (14 %)	44 (14 %)
10 -14 years	30 (14 %)	63 (20 %)
15 + years	126 (59 %)	165 (53 %)
*Insulin treatment type, N (%)*		
Basal-only therapy	13 (6 %)	76 (23 %)
Basal-bolus therapy	135 (61 %)	162 (49 %)
Other insulin types	74 (33 %)	93 (28 %)
*Duration of insulin treatment, N (%)*		
Average in years (SD)	18.5 (13.5)	8.3 (6.7)
< 2 years	13 (6 %)	38 (12 %)
2–5 years	27 (13 %)	91 (29 %)
5–9 years	25 (12 %)	61 (20 %)
10 + years	149 (70 %)	122 (39 %)
*Mean HbA1c*		
mean mmol/mol (SD);	55.4 (11.5)	61.3 (18.8)
NGSP %, (SD)	7.2 (1.1)	7.8 (1.7)
Medical complications^a^, none, N (%)	152 (68 %)	125 (38 %)

*BMI* Body mass index, *HbA1c* Haemoglobin A1c (glycosylated haemoglobin), *NGSP* National Glycohaemoglobin Standardisation Programme, *SD* Standard deviation

^a^Questionnaire options for medical complications included: None, Eye problems, Neuropathy, Cardiovascular disease, Renal disease, Amputations, Other (please specify)

The mean annual self-reported frequencies of NSHE were 85 for T1DM, 22 for T2DM, 15 for T2BOT, 28 for T2BB and 19 for T2O. Analysing results only from those respondents who experienced a NSHE during the recall period, annual NSHE rates were 100 in T1DM and 39 in T2DM (ranging from 33 in T2O to 43 in T2BB) (Table 2). The proportion of NSHEs which occurred at night-time was 19 % for T1DM and 21 % for T2DM respondents. After excluding respondents who did not experience any nocturnal NSHEs during the study period, the proportion of events occurring at night-time increased to 23 % (*n* = 29) in T1DM and 39 % (*n* = 20) in T2DM (Table 3).

**Table 2:** Self-reported, recalled rates of hypoglycaemic events (daytime and nocturnal combined)

	*T1DM (n* ***=*** *222; 716 pw)*	*T2DM*			
		*T2DM (n* ***=*** *331; 1,057 pw)*	*T2BOT (n* ***=*** *76; 248 pw)*	*T2BB (n* ***=*** *162; 532 pw)*	*T2O (n* ***=*** *93; 277 pw)*
Annual calculated NSHE rates (52 weeks), mean	84.6	22.5	15.1	27.8	19.0
Patients who experienced ≥ 1 NSHE in study period, n (%)	177 (80 %)	174 (53 %)	28 (37 %)	99 (61 %)	47 (51 %)
	*T1DM (n = 177; 605 pw)*	*T2DM (n = 174; 605 pw)*	*T2BOT (n = 28; 103 pw)*	*T2BB (n = 99; 343 pw)*	*T2O (n = 47; 159 pw)*
NSHEs/year, mean	100.1	39.3	36.3	43.1	33.0
Nocturnal NSHEs/year, mean (% of all NSHEs)	19.1 (19 %)	8.1 (21 %)	8.1 (22 %)	9.6 (22 %)	4.9 (15 %)

Base: All respondent-weeks reported; includes all respondents regardless of whether completing all four questionnaires

*NSHE* Non-severe hypoglycaemic event, *SD* Standard deviation, *T1DM* Type 1 diabetes mellitus, *T2BB* Type 2 diabetes mellitus respondents receiving basal bolus therapy/short and long acting insulin, *T2BOT* Type 2 diabetes mellitus respondents receiving basal only therapy/long acting insulin only, *T2O* Type 2 diabetes mellitus respondents receiving other therapy (e.g. mixed insulin)

**Table 3:** Self–reported, recalled rates of non-severe hypoglycaemic events (nocturnal only)

*All respondents (1773 respondent-week records from 553 respondents)*					
	*T1DM (n = 222; 716 pw)*	*T2DM*			
		*T2DM (n = 331; 1,057 pw)*	*T2BOT (n = 76; 248 pw)*	*T2BB (n = 162; 532 pw)*	*T2O (n = 93; 277 pw)*
Nocturnal NSHEs/year, mean (% of al NSHEs)	16.1 (19 %)	4.6 (21 %)	3.4 (22 %)	6.2 (22 %)	2.8 (15 %)
Patients who experienced ≥ 1 nocturnal NSHE in study period, n (%)	110 (50 %)	72 (22 %)	10 (13 %)	48 (30 %)	14 (15 %)
*Respondents who experienced ≥ 1 nocturnal NSHE in the study period*					
	*T1DM (n = 110; 392 pw)*	*T2DM (n = 72; 48 pw)*	*T2BOT (n = 10; 40 pw)*	*T2BB (n = 48; 162 pw)*	*T2O (n = 14; 46 pw)*
NSHEs/year, mean	127.3	50.3	54.6	51.7	41.9
Nocturnal NSHE/year, mean	29.4 (23 %)	19.7 (39 %)	20.8 (38 %)	20.2 (39 %)	17.0 (41 %)

Base: All respondent-weeks reported; includes all respondents regardless of whether completing all four questionnaires

*NSHE* Non-severe hypoglycaemic event, *SD* Standard deviation, *T1DM* Type 1 diabetes mellitus, *T2BB* Type 2 diabetes mellitus respondents receiving basal bolus therapy/short and long acting insulin, *T2BOT* Type 2 diabetes mellitus respondents receiving basal only therapy/long acting insulin only, *T2O* Type 2 diabetes mellitus respondents receiving other therapy (e.g. mixed insulin)

Self-reported mean annual SHE frequencies were 0.7 for T1DM and 0.2 for T2DM (0.1 for T2BOT, 0.2 for T2BB and 0.2 for T2O).

The proportion of respondents who are reportedly ‘aware’ of hypoglycaemic symptoms was 48 % in T1DM and 57 % in T2DM respondents. Respondents who reported impaired awareness comprised 44 % of T1DM respondents and 31 % of T2DM respondents, with 7 and 12 % being unaware, respectively (Table 4). For T1DM respondents, there was a trend for increased NSHE and SHE rates with decreasing awareness, however this was not significant (data not shown). NSHE rates for T2DM respondents were comparable regardless of the level of awareness (data not shown). There may also be an association between HbA1c levels and awareness, however, opposing trends are observed according to diabetes type. In T1DM respondents, low HbA1c corresponded with reduced unawareness (6.7 % in unaware versus 7.4 % in aware patients), however in T2DM higher HbA1c corresponded with reduced awareness (8.4 % in unaware versus 7.6 % in aware patients).

**Table 4:** Self-reported respondent awareness of hypoglycaemia

**All respondents who have previously experienced a NSHE† (** ***n*** **=** **396** **)**		**T1DM (n** **=** **189)**	**T2DM**			
			All T2DM (n = 207)	T2BOT (*n* = 35)	T2BB (n = 115)	T2O (n = 57)
Can you feel when your blood sugar is low? N (%)	Always aware	91 (48 %)	119 (57 %)	15 (43 %)	69 (60 %)	35 (61 %)
	Impaired awareness	84 (44 %)	64 (31 %)	10 (29 %)	38 (33 %)	16 (28 %)
	Unaware	14 (7 %)	24 (12 %)	10 (29 %)	8 (7 %)	6 (11 %)

Hypoglycaemia ‘awareness’ relates to the respondents’ self-reported ability to recognise the physical symptoms indicating the onset of a hypoglycaemic event. †Base: all respondents that have previously experienced a NSHE at any point (not just in study recall period)

*NSHE* Non-severe hypoglycaemic event, *T1DM* Type 1 diabetes mellitus, *T2BB* Type 2 diabetes mellitus respondents receiving basal bolus therapy/short and long acting insulin, *T2BOT* Type 2 diabetes mellitus respondents receiving basal only therapy/long acting insulin only, *T2DM* Type 2 diabetes mellitus, *T2O* Type 2 diabetes mellitus respondents receiving other therapy (e.g. mixed insulin)

Overall, 67 % of T1DM and 49 % of T2DM respondents rarely or never inform their general practitioner (GP)/specialist about hypoglycaemic events. When respondents were asked about topics discussed during GP/specialist consultations, 18 % of T1DM and 20 % of T2DM respondents reported that their GP/specialist did not ask about hypoglycaemia during routine appointments (Table 5). NSHE rates were similar regardless of the level of patient–physician communication (data not shown).

**Table 5:** Patient–physician communication of hypoglycaemia

	*T1DM (n = 189)*	*T2DM*				
		*All T2DM (n = 207)*	*T2BOT (n = 35)*	*T2BB (n = 115)*	*T2O (n = 57)*	
Proportion of respondents who rarely/never inform their GP/specialist about NSHEs, N (%)	126 (67 %)	101 (49 %)	15 (43 %)	56 (49 %)	30 (53 %)	
	*T1DM (n = 222)*	*All T2DM (n = 331)*	*T2BOT (n = 76)*	*T2BB (n = 162)*		*T2O (n = 93)*
GP/specialist did not ask about hypoglycaemia during routine appointments	18 %	20 %	14 %	25 %		16 %

*NSHE* Non-severe hypoglycaemic event, *SD* Standard deviation, *T1DM* Type 1 diabetes mellitus, *T2BB* Type 2 diabetes mellitus respondents receiving basal bolus therapy/short and long acting insulin, *T2BOT* Type 2 diabetes mellitus respondents receiving basal only therapy/long acting insulin only, *T2DM* Type 2 diabetes mellitus, *T2O* Type 2 diabetes mellitus respondents receiving other therapy (e.g. mixed insulin)

^a^Base: all respondents that have previously experienced a NSHE at any point (not just in study recall period)

^b^Base: All respondents completing wave 1 (*n* = 396)

**Fig. 1:**
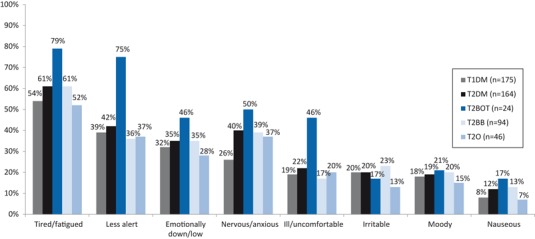
Negative health-related impacts of NSHEs. From left to right: most common response to least common response, based on combined results from Type 1 diabetes mellitus and Type 2 diabetes mellitus. *NSHE* Nonsevere hypoglycaemic event, *T1DM* Type 1 diabetes mellitus, *T2BB* Type 2 diabetes mellitus respondents receiving basal bolus therapy/short and long acting insulin, *T2BOT* Type 2 diabetes mellitus respondents receiving basal only therapy/long acting insulin only, *T2DM* Type 2 diabetes mellitus, *T2O* Type 2 diabetes mellitus respondents receiving other therapy (e.g. mixed insulin)

Respondents reported negative health-related impacts following their last NSHE, including feeling tired/fatigued (following 54 and 61 % of NSHE in T1DM and T2DM, respectively), less alert (39 and 42 % of NSHE in T1DM and T2DM) and ill/uncomfortable (19 and 22 % of NSHE in T1DM and T2DM). Respondents’ emotional wellbeing was also affected, with NSHE resulting in feeling emotionally low (following 32 and 35 % of NSHE in T1DM and T2DM, respectively), anxious/nervous (26 and 40 % in T1DM and T2DM) and moody (18 and 19 % in T1DM and T2DM) (Fig. 1). The number of hours negative feelings lasted for was greater in T2DM respondents (7 h in T2DM versus 4 h in T1DM). Events which occurred at night-time had a longer lasting impact than events during the day: 7 versus 4 h in T1DM and 8 versus 6 h in T2DM. NSHEs also impacted upon respondents’ daily routine. The three most common impacts were reduced energy levels, daytime sleeping and difficulty concentrating. These overall findings were reflected in the specific results for respondents with T1DM or T2DM. T1DM respondents reported having less energy than usual following 34 % (daytime) and 50 % (nocturnal) of NSHE, with 25 % (daytime) and 23 % (nocturnal) NSHE resulting in daytime sleeping. T2DM respondents reported similar trends with 56 % of daytime and 32 % of nocturnal NSHE resulting in reduced energy levels and 56 % (daytime) and 29 % (nocturnal) of NSHE leading to daytime sleeping.

NSHEs reported during the study period resulted in increased use of healthcare resources (Table 6) [16]. Over the seven days following a NSHE, blood glucose test-strip use increased by a mean of 4.1 (13 %) in T1DM and 3.7 (17 %) in T2DM respondents. In T1DM and T2DM respondents combined, 6 % of NSHE led respondents to contact a HCP (Table 6). A greater proportion of respondents with T2DM contacted a HCP, regardless of the time of day that the event occurred (daytime NSHE: 10 %, nocturnal NSHE 9 %). In employed T1DM respondents (*n* = 143), 8 % of NSHE led to lost work time with approximately 3.0 h work time lost per event. In employed T2DM respondents (*n* = 74), 14 % of NSHE led to lost work time, with approximately 4.3 h work time lost per event (Table 6).

**Table 6:** Direct and indirect economic impacts of hypoglycaemic events

*Last NSHE across all respondents*	T1DM	T2DM
NSHE resulting in contact with HCP		
Overall, % (n)	3 (12)	10 (33)
Daytime NSHE, % (n)	3 (11)	10 (27)
Nocturnal NSHE, % (n)	1 (1)	9 (6)
Self-reported number of BGM tests use in the average week, mean	30.4	22.2
*Mean increase in BGM use in the 7 days following a NSHE*		
Overall	4.1	3.7
Daytime NSHE	3.5	3.8
Nocturnal NSHE	5.9	3.1
*Last NSHE from employed respondents¥*		
*NSHE leading to lost work time, %*		
Overall, % (n)	8 (23)	14 (12)
Daytime NSHE, % (n)	8 (21)	13 (8)
Nocturnal NSHE, % (n)	4 (2)	18 (4)
*Mean work time lost after a NSHE (in respondents who lost work time), mins*		
Overall	181.9	256.5
Daytime NSHE	185.0	131.3
Nocturnal NSHE	150.0	507.0
NSHE leading to self-reported inability to complete a work task in a timely manner, %	42	38
NSHE resulting in self-reported difficulty concentrating at work, %	27	28

*BGM* blood glucose measurement, *HCP* healthcare professional, *Mins* Minutes, *N/A* Not applicable, *NSHE* Non-severe hypoglycaemic event, *SHE* severe hypoglycaemic event, *T1DM* Type 1 diabetes mellitus, *T2DM* Type 2 diabetes mellitus

## Discussion

This study evaluates the real world frequency of NSHEs and SHEs in people with T1DM and insulin-treated T2DM in Austria. In addition, it provides insight into hypoglycaemic awareness, patient–physician communication and the health-related and economic impacts linked to non-severe events.

The frequency of NSHEs in T1DM respondents in Austria (1.6 per week), is slightly lower than in three previously conducted studies in Northern and Central Europe, which reported NSHE frequencies of 1.8, 2.0 and 2.2 per respondent, per week [15, 20, 21]. However, event rates in T2DM respondents (0.4 per week) are similar to those reported in a prospective single-centred study in Scotland (0.3 per week) [8]. The Austrian NSHE rate is generally similar to the mean rate previously reported for Europe (based on seven countries including Austria) [15, 16], across all patient groups (85 versus 91 in T1DM and 15–28 versus 20–35 in T2DM) [15, 16]. The proportion of events which occur at night-time in Austria (19 % in T1DM and 15–22 % in T2DM) is also comparable to the results across all European countries studied (22 % in T1DM and 22–32 % in T2DM) [15].

In Austria, the self-reported NSHE rate in T1DM respondents is four times greater than the rates reported by T2DM respondents. It has previously been shown that the risk of hypoglycaemia in people with insulin-treated T2DM increases with increasing diabetes duration [8], and Henderson et al. [22] reported that NSHE frequency among people with T2DM only reaches the same level as in people with T1DM after 10 years of insulin use in T2DM [22]. In the present study, only 39 % of respondents with T2DM had received insulin for over 10 years (compared to 70 % of respondents with T1DM), which may help to explain the lower frequency of NSHEs. In T2DM the frequency of NSHE also varies according to treatment regimen, although this is expected due to the different types of insulin coverage [23].

Although we report a mean annual NSHE rate of 85 in T1DM and 22 in T2DM patients in Austria, sub-analyses suggest that some patients are more susceptible to hypoglycaemia. Over a third (37 %) of all respondents did not experience any NSHEs during the four week study period; a sub-analysis excluding these respondents increases the mean annual NSHE rate by 18 % in respondents with T1DM, and by 77 % in respondents with T2DM (ranging from a 54 % increase in T2BOT to 140 % in T2BOT respondents). Looking specifically at nocturnal events, although the mean annual rate was 16 in T1DM and 5 in T2DM respondents, 50 % of respondents with T1DM and 78 % of T2DM respondents did not experience any nocturnal NSHEs during the study. A sub-analysis of the respondents who reported at least one nocturnal event resulted in nearly double the nocturnal NSHE rate in T1DM and increased it 4-fold in T2DM respondents. These results suggest an increased risk of hypoglycaemia in patients with a history of events, a finding which has been reported previously [24–30]. As some patients may not regularly experience NSHEs (one third of this study population), or only experience them during the day-time (two thirds of this study population), the real world burden in those that have regular hypoglycaemic events may be higher than previously reported. It is important to consider that the current results only relate to a four-week period, so further research is warranted to better understand this issue.

In the present study, we investigated patients’ self-reported ability to recognise the symptoms of hypoglycaemia. There is no consensus on how to classify awareness, however our method benefits from the use of three categories (instead of two—aware/unaware—as in the Clarke [31] and Gold [32] methods), which enables identification of the gradual loss of awareness. In addition, it is the only method proven to perform similarly across language barriers [33]. The proportion of respondents in Austria reportedly ‘aware’ of hypoglycaemic symptoms was higher than the average across Northern and Central Europe in both T1DM (48 % versus 36 %) and T2DM respondents (43–61 % versus 36–51 %) [15]. Despite higher awareness levels in Austria compared to the rest of Northern and Central Europe, a substantial proportion of our study population (47 %) had impaired awareness or unawareness. Impaired awareness has been linked to reduced adherence to recommended changes in insulin regimen [34], and has been reported as the most important risk factor for severe hypoglycaemia [35]. A number of previous studies have shown a statistically significant (*p* < 0.05) increase in SHEs in patients with reduced awareness [34, 36–39]. In line with this, our study showed a trend (although not statistically significant) for increased SHE and NSHE rates with reduced levels of awareness (in respondents with T1DM). In the overall European study, these trends were statistically significant (*p* < 0.05) [16]. This could be explained by unaware respondents not taking preventative action to stop the onset of hypoglycaemia, because they are unable to recognise the symptoms of low blood sugar. Additionally, this inability may cause respondents to overcompensate by testing their blood glucose more frequently, resulting in the identification of more events.

We also found that a high proportion of respondents in Austria were reluctant to discuss their hypoglycaemia with their GP/specialist (67 % of T1DM and 49 % of T2DM; comparable to the mean percentages across Northern and Central Europe) [15]. This may be due to wider factors such as a fear of losing driving privileges [11], impacts in the work environment, or concerns that their GP may think they have poor control of their diabetes.

Unsurprisingly, NSHE were associated with reduced physical and emotional well-being, regardless of the time of day the NSHE occurred. The negative emotional impact of NSHEs was comparable across diabetes types; tiredness/fatigue and reduced alertness were the most commonly reported effects in both T1DM and T2DM respondents. This supports a previous study in which patients reported that hypoglycaemia affects their daily life and causes anxiety [10]. The health-related impact of hypoglycaemia has been further confirmed using the EQ-5D and SF-36; a study of diabetes patients in the UK reported that as the frequency and severity of hypoglycaemia increased, quality of life and health-related utility decreased [7].

In our study, patients increased their self-monitoring of glucose levels in response to a NSHE. Whilst this is an appropriate adaptive behaviour which may help prevent new events in the short-term [11], it increases healthcare resource use (i.e. increase in BGM test-strip consumption) and is therefore associated with a cost burden. This burden could be alleviated if NSHE frequency was reduced, through improved awareness and recognition of events. An additional contributor to the direct cost burden of NSHEs in Austria is patient contact with physicians to report their event. NSHE also present an indirect cost burden in Austria through lost work time. This is reported in further detail by Geelhoed-Duijvestijn et al. [16].

Limitations of this study should be considered and have been discussed previously by Östenson et al. [15] and Geelhoed-Duijvestijn et al. [16]. Respondent demographics show that 6 % of Austrian respondents with T1DM were receiving basal-only insulin, however this formulation should only be used in patients with T2DM, and therefore we presume that most of these respondents incorrectly reported their diabetes type as T1DM. Given that T2DM is associated with fewer hypoglycaemic events, our study may underestimate the true frequency of events among respondents with T1DM.

Secondly, the survey relies on respondents ability to recall their NSHEs frequency over the preceding 7-day period, which might have introduced a bias related to the interpretation of symptoms. However, a previous study reported that a respondent’s recall of NSHEs during the previous week was not significantly different from the prospective recording of events over the same time period [14]. The recruitment method, which required having an email address and used online panels to locate patients, could have introduced selection bias. However, the internet penetration rate in Austria is higher than the average across Europe, based on data from 2012 (80 % in Austria compared to 73 % across the European Union and 64 % across Europe) [40] and recruitment was done via broad panels reflective of the general population. As respondents were not informed that the survey was about hypoglycaemia prior to enrolling, there is no reason to suggest any selection bias towards people struggling with hypoglycaemia in the first wave of the study. However since the response rates for waves of the study diminished (82, 72 and 67 % of respondents completed wave two, three and four respectively), we cannot rule out the possibility that later waves were completed by respondents who had more experience of hypoglycaemic events.

Despite these limitations, this study provides important real-world rates of hypoglycaemia in Austria, both during the day and at night-time, addressing the lack of data available for this population. Many patients are unable to recognise the symptoms of a hypoglycaemic event, and also express a reluctance to discuss their hypoglycaemia with physicians. The importance of improving glycaemic control is evidenced by the negative impact on healthcare resource use and work productivity in Austria that results from even non-severe events.
